# Changes in the gut microbiota in mice exposed to chronic intermittent hypoxia

**DOI:** 10.1099/jmm.0.002069

**Published:** 2025-09-22

**Authors:** Man-Lu Lu, Jing-Lin Wu, Ji-Wei Zhu, Lu Liu, Ming-Zhen Li, Yan Yu, Lei Pan

**Affiliations:** 1 Department of Respiratory and Critical Care Medicine, Binzhou Medical University Hospital, Binzhou 256603, PR China

**Keywords:** gut microbiota, intermittent hypoxia, normoxia, obstructive sleep apnoea

## Abstract

**Introduction.** Obstructive sleep apnoea syndrome (OSAS) is characterized by chronic intermittent hypoxia (CIH), which contributes to systemic complications, including metabolic and gastrointestinal disorders. Emerging evidence suggests a critical role of the gut microbiota in mediating these effects; however, the impact of CIH on the gut microbiota remains poorly understood.

**Gap Statement.** While CIH is associated with systemic metabolic dysfunction, the specific alterations in gut microbiota composition and function induced by CIH remain understudied. Filling this knowledge gap could elucidate microbiota-mediated mechanisms of OSAS pathogenesis and identify therapeutic targets.

**Aim.** To investigate the effects of CIH on the gut microbiota structure and functional pathways in a mouse model of OSAS.

**Methodology.** Male C57BL/6 mice were exposed to normoxia (NM) or CIH conditions for 6 weeks. Faecal samples were collected via stress defecation before intervention (NM0 and CIH0 groups) and after 6 weeks (NM6 and CIH6 groups). Gut microbiota composition was assessed using 16S rRNA gene sequencing, and functional potential was predicted via Phylogenetic Investigation of Communities by Reconstruction of Unobserved States 2.

**Results.** A total of 40 faecal samples (10 mice/group) were analysed. No significant differences in microbiota composition, alpha diversity or beta diversity were observed between groups before intervention. CIH significantly altered gut microbiota composition and abundance. At the genus level, *Bacteroides* abundance increased (rank-biserial=0.558, *P*=0.014) in CIH6 mice, while *Bifidobacterium* (Cohen’s d=1.779, *P*=0.002), *Helicobacter* (rank-biserial=0.609, *P*=0.007) and *Prevotella* (rank-biserial=0.541, *P*=0.0173) decreased. Linear discriminant analysis effect size (LEfSe) and random forest model analyses identified these genera as key discriminators of microbiota composition. Kyoto Encyclopedia of Genes and Genomes functional prediction revealed 28 significantly altered tertiary metabolic pathways in CIH6 mice, including biotin, lipoic acid, beta-alanine and cyanoamino acid metabolism.

**Conclusion.** CIH induces gut microbiota dysbiosis, disrupts short-chain fatty acid-producing bacteria and impacts multiple metabolic pathways. This study provides evidence linking gut microbiota alterations to OSAS pathogenesis and offers a theoretical foundation for targeting the microbiome as a potential therapeutic strategy for CIH-related disorders.

## Data Summary

The number of sequences obtained per sample is detailed in supplementary Table S1.

## Introduction

Obstructive sleep apnoea syndrome (OSAS) is a sleep-disordered breathing condition characterized by recurrent collapse of the upper airway. Obesity, craniofacial changes and altered upper airway neuromuscular function are significant factors contributing to upper airway collapse. It is characterized by chronic intermittent hypoxia (CIH), hypercapnia and chronic sleep fragmentation [1,2]. A large French population-based cohort has shown that the prevalence of OSAS in the general population is 20.9%, with a higher prevalence in men than in women, and despite one-fifth of the population exhibiting symptoms indicating a high risk of OSAS, only 3.5% of the patients with sleep apnoea receive treatment [3]. It is evident that OSAS has become one of the common conditions in the general population. However, it receives low priority from the public.

The gut microbiota refers to a microbial community that inhabits the intestine, primarily composed of bacteria, archaea, fungi and viruses [4]. This microbial community is predominantly composed of four major bacterial phyla: Firmicutes, Bacteroidetes, Actinobacteria and Proteobacteria [5,6]. The composition and structure of the gut microbiota are influenced by multiple factors [7], with CIH being recognized as one of the potentially critical determinants [8]. Prolonged exposure to CIH can result in comprehensive alterations in the composition of the gut microbiota and the circadian rhythm of the intestinal environment, and these changes in intestinal physiology may contribute to the pathophysiological progression of diseases such as atherosclerosis, type 2 diabetes and colorectal cancer [9,11]. Badran *et al*. found that transplantation of gut microbiota from CIH-exposed mice into the intestines of naive mice resulted in reproduction of adverse cardiovascular reactions observed in CIH mice, while supplementation with probiotics alleviated cardiovascular damage in naive mice [12]. Therefore, it is essential to investigate the alterations in the composition and structure of gut microbiota, as well as related pathways in the CIH environment, to clarify the relationship between CIH and gut microbiota. This study simulated the CIH state of OSAS in mice, revealing the structural characteristics of the gut microbiota and the alterations in metabolic pathways in the OSAS mouse model. Moreover, it examined the effects of CIH on the gut microbiota of mice, aiming to propose innovative intervention strategies and research directions for the prevention and treatment of OSAS and its co-morbidities.

## Methods

### Experimental animal

Twenty healthy 5-week-old male C57BL/6 J mice, specific-pathogen-free (SPF) grade, weighing 20±2 g, were obtained from Gempharmatech Co., Ltd. [animal licence number: SCXK (Su) 2018-0008]. All mice were housed in an SPF animal facility with a temperature maintained at ~25 °C and a relative humidity of about 50%. The mice were subjected to a 12 h light-dark cycle, and they were given free access to water and food throughout the feeding period. After acclimatizing to the new environment for 1 week, 6-week-old mice were randomly assigned to two groups: the normoxia (NM) group (*n*=10) and the CIH group (*n*=10). To mitigate the potential confounding effects of shared microbiome due to co-housing and to minimize experimental batch effects, the mice in the CIH group were housed in two separate cages with an equal number of animals in each cage. Similarly, the NM group mice were also distributed into two cages using the same housing strategy. This separated housing arrangement was maintained consistently throughout the entire experimental period. The NM0 and CIH0 groups served as the pre-intervention groups, while the NM6 and CIH6 groups served as the post-intervention groups. This research protocol was approved by the Committee on Animal Research and Ethics of the Affiliated Hospital of Binzhou Medical College (approval number: 20221014-10), and all experiments were conducted following the 3R principle.

### Construction of the CIH animal model in mice

Mice were placed into a hypoxic simulation chamber (BioSpherix Oxycycler A84, USA) daily from 8 : 00 am to 4 : 00 pm, exposing them to a CIH environment for 8 h daily for 6 weeks. The operational procedure of the hypoxia simulation chamber was as follows: nitrogen was infused into the chamber for 85–90 s, reducing the oxygen content from 21% (normoxic state) to 7% (hypoxic state), which lasted for 25–30 s. Subsequently, oxygen was introduced into the chamber for 45–50 s, restoring the oxygen content to 21% (normoxic state), lasting for 25–30 s. This completed one cycle of CIH, with subsequent cycles repeating in this manner [13]. This hypoxia/reoxygenation alternation effectively simulates CIH events in patients with OSAS.

### Collection and preservation of mouse faecal samples

In a controlled experimental setting, fresh faecal samples from mice can be obtained by utilizing their natural defecation response to external stressors, such as restraint. The experimenter grasps the ears and the skin on the neck of the mouse firmly. Subsequently, the tail was gently lifted, and the fresh faecal pellets were directly obtained after excretion. Three to five faecal pellets were collected in the sterilized centrifuge tubes. After collection, the centrifuge tubes containing faeces were immediately placed into liquid nitrogen, and the samples were transferred to −80℃ for cryopreservation immediately afterward. A total of 40 fresh faecal samples were collected from 2 groups of mice before and after intervention.

### Determination of faecal microbiota

#### DNA extraction and PCR amplification of the 16S rRNA gene of gut microbiota

DNA was extracted using the OMEGA Soil DNA Kit (M5636-02) (Omega Bio-tek, Norcross, GA, USA). Total genomic DNA was extracted according to the manufacturer’s instructions and stored at −20 °C. The integrity of the extracted DNA was determined by 0.8% agarose gel electrophoresis. DNA concentration and purity were measured using a NanoDrop NC2000 spectrophotometer (Thermo Fisher Scientific, Waltham, MA, USA). PCR amplification of the highly variable region V3-V4 of bacterial 16S rRNA was performed using a forward primer 338F (5′-ACTCCTACGGGAGGCAGCA-3′) and a reverse primer 806R (5′-GGACTACHVGGGTWTCTAAT-3′) [14]. The total system of the PCR reaction mixture was 25 µl. The PCR components were 0.25 µl of Q5 High-Fidelity DNA polymerase, 5 µl of Reaction Buffer (5×), 5 µl of High GC Buffer (5×), 2 µl of dNTP (10 mM), 2 µl of DNA template, 1 µl (10 µM) of each forward and reverse primer and 8.75 µl of ddH2O. The PCR negative control group was set up in parallel, containing all components of the PCR reaction system, except that the DNA template was replaced with sterile nuclease-free water. After configuring the required components for the PCR reaction, set the PCR thermal cycle conditions: 98 °C for 30 s (initial denaturation), 98 °C for 15 s (denaturation), 50 °C for 30 s (annealing) and 72 °C for 30 s (extension), repeated for 25–27 cycles to accumulate a large number of amplified DNA fragments. Finally, the reaction was maintained at 72 °C for 5 min to ensure complete extension of the product. The PCR amplification products were purified with Vazyme VAHTS^™^ DNA Clean Beads (Vazyme, Nanjing, China) and quantified using the Quant-iT PicoGreen dsDNA detection kit (Invitrogen, Carlsbad, CA, USA). The amplicons were pooled in equal amounts.

#### Library construction and Illumina MiSeq sequencing

Sequencing libraries were established using the TruSeq Nano DNA LT Library Prep Kit from Illumina, and the 16S rRNA V3-V4 region was subjected to paired-end 2×250 bp sequencing on the Illumina MiSeq platform using MiSeq Reagent Kit v3 (600 cycles) at Shanghai Personal Biotechnology Co., Ltd (Shanghai, China)

#### Sequencing data processing

The acquired sequences were processed using QIIME 2. Raw sequence data were demultiplexed using the demux plugin followed by primers cutting with the cutadapt plugin. Sequences that do not match primers were discarded and then quality-filtered, denoised, merged and chimaera-removed using the Divisive Amplicon Denoising Algorithm 2 (DADA2) plugin [15]. Each de-duplicated sequence (equivalent to clustering with 100% similarity) generated after processing with DADA2 was referred to as an amplicon sequence variant (ASV), or feature sequence, and high-quality sequences were finally obtained. ASVs with a total sequence count of only one were removed. Alpha and beta diversity analyses of the obtained sequences were conducted on the QIIME 2 platform. The Shannon index was used to assess the alpha diversity of the samples to explore the composition of the faecal microbiota. Beta diversity was analysed by plotting principal coordinate analysis (PCoA) using Bray–Curtis distances. LEfSe was used to explore the differential gut microbiota among the groups, with the logarithmic linear discriminant analysis (LDA) score cutoff of 2.5. On the QIIME 2 platform, the classify-sklearn algorithm was employed to perform taxonomic annotation based on the Greengenes database. Random forest analysis was performed using QIIME 2. Based on the absolute abundance table of taxonomic units at the genus level, the ‘classify_samples_ncv’ function in q2-sample-classifier was invoked to perform random forest analysis and nested stratified cross-validation. Considering that the maximum number of samples in a single group in this study was ten, fivefold cross-validation was carried out. On the Phylogenetic Investigation of Communities by Reconstruction of Unobserved States 2 (PICRUSt2) platform, MAFFT was employed to align the non-singleton ASVs that were generated after DADA2 processing with the reference sequences. Subsequently, FastTree 2 was utilized to construct a phylogeny based on the alignment results. The Castor hidden state prediction algorithm was used to infer the nearest sequence species of the feature sequences, and then, the gene family copy numbers of these feature sequences were obtained. The predicted metagenomes were then mapped to the Kyoto Encyclopedia of Genes and Genomes (KEGG) database, and functional classification was performed based on KEGG homology [16]. The number of sequences obtained per sample is detailed in supplementary table S1, available in the online Supplementary Material.

#### Statistical analysis

SPSS version 26.0 (IBM Corp, Chicago, USA) was utilized for statistical analysis of the data. Normality was examined using the Shapiro–Wilk test (*P*>0.05 indicating normality), and homogeneity of variances was assessed using Levene’s test (*P*>0.05, indicating that the variances were homogeneous). Quantitative data conforming to normality and homogeneity of variance were compared between the two groups using either Student’s t-test or paired sample t-test, while non-normally distributed quantitative data were compared between the two groups using either Wilcoxon rank-sum test or Wilcoxon signed-rank test. A *P* value<0.05 was considered statistically significant.

## Results

### Analysis of sequence results

A total of 40 faecal samples were included in this study. Among them, ten samples each from the NM0 and CIH0 groups served as baseline, while ten samples each from the NM6 and CIH6 groups represented the post-intervention groups. High-throughput sequencing was conducted on the V3-V4 region of the bacterial 16S rRNA gene in these faecal samples. The sequencing results revealed a sequence length distribution ranging from 50 to 439 bp, with a total of 1,882,323 valid sequences and a cumulative length of 788,596,113 bp, and the average sequence length was 418.948 bp, indicating that the sequencing data in this study sufficiently covered the majority of gut microbiota.

### Quality evaluation of gut microbiota sequencing

**Fig. 1.**
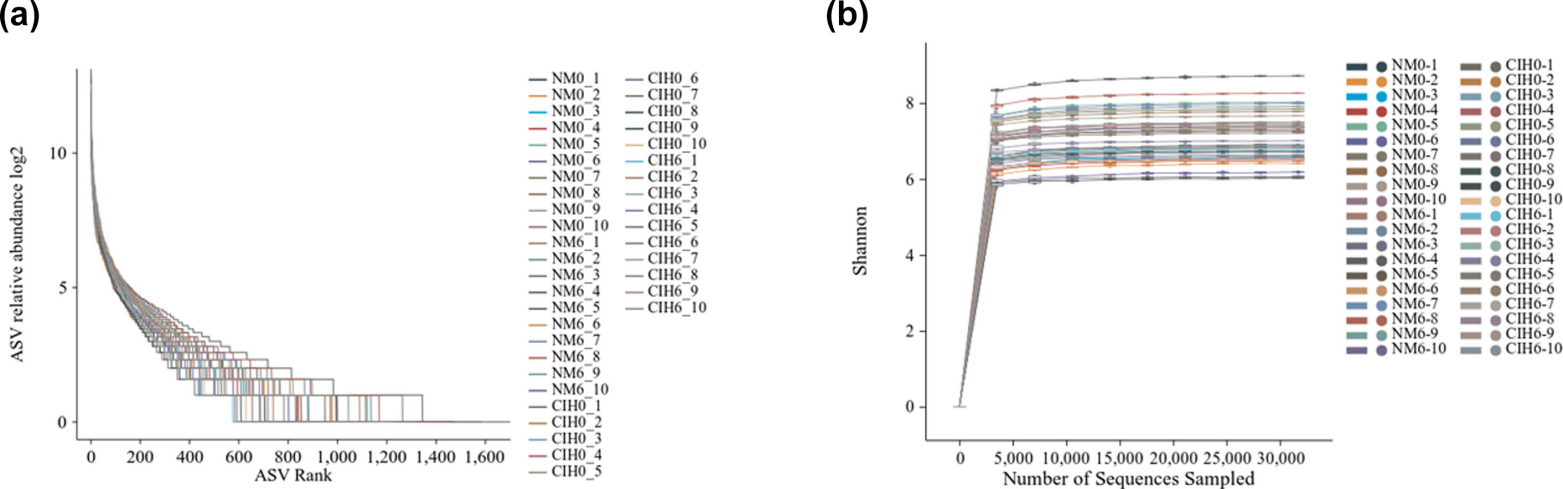
(**a**) Rank abundance curve of faecal sample microbiota. The relative abundance of each ASV in each sample was calculated. Specifically, subsequently, all ASVs of each sample were sorted in descending order according to their relative abundances. For the construction of the rank abundance curve, the rank of each ASV, starting from 1 for the ASV with the highest relative abundance, was set as the X-axis (labelled as ‘ASV Rank’), and the relative abundance of each ASV was converted by log2 and then set as the Y-axis (labelled as ‘ASV relative abundance log2’). (**b**) Rarefaction curves based on the Shannon index. In this study, we sampled subsets of reads from high-quality reads that had been filtered to remove low-quality bases, adapter sequences and potential contaminants. For each subset of reads, they were first processed through quality filtering, denoising and dereplication. Then, the high-quality sequences in each subset were clustered into ASVs. The Shannon index was calculated based on the obtained ASVs. Using the Shannon index as the ordinate and the number of sequences sampled as the abscissa, we constructed rarefaction curves.

The rank abundance curve (Fig. 1a) demonstrated that as the ASV rank of faecal samples increases, the curve tends to flatten, and the span on the horizontal axis gradually widens, indicating that the richness and evenness of microbiota in each group of samples reached a high level. In this study, rarefaction curves were constructed based on the Shannon diversity index. The results, as shown in Fig. 1(b), indicated that when the number of randomly sampled sequences reached ~5,000, the curve gradually levelled off. This indicated that further increasing the sequencing depth would not lead to the discovery of a large number of previously undetected ASVs. This result demonstrates that the sequencing depth in this study is adequate to capture the vast majority of the microbial diversity information in the samples, and the obtained sequencing data can be effectively utilized for subsequent analyses.

### Diversity analysis of the gut microbiota

**Fig. 2.**
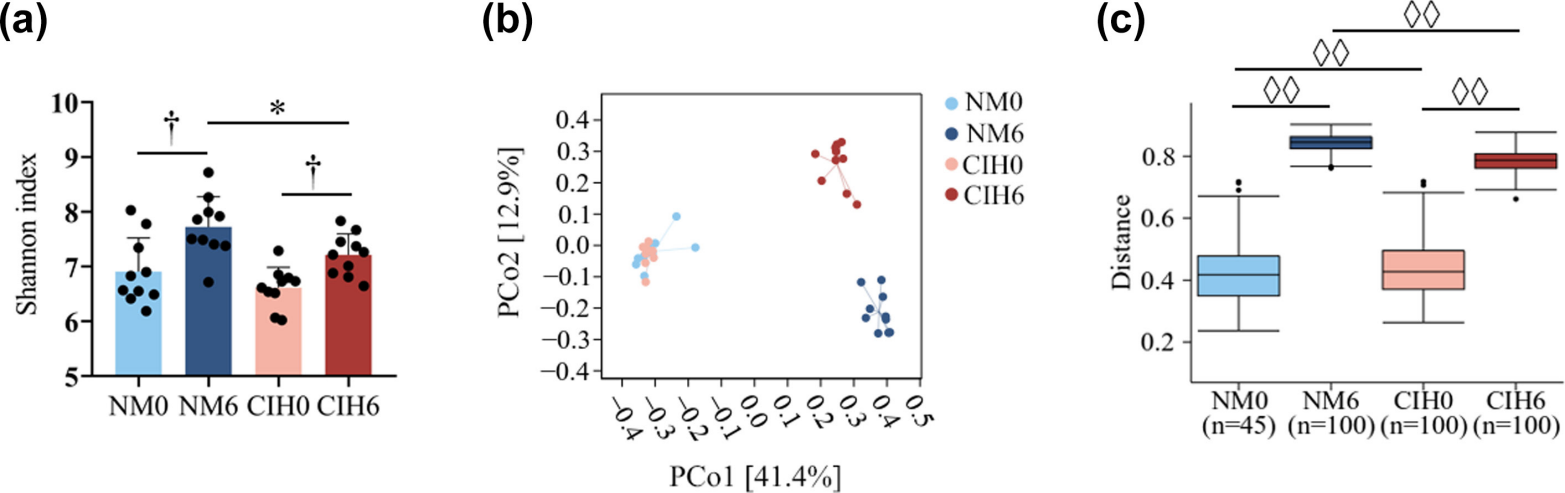
(**a**) Comparison of the alpha diversity of gut microbiota among different groups, presented as the Shannon index reflecting species evenness. Statistical significance between the NM group and CIH group was assessed by Student’s t-test (^*^P<0.05), while differences between the 0-week and 6-week groups were evaluated using paired sample t-test (^†^*P*<0.05). (**b**) Comparison of the beta diversity of gut microbiota among different groups, presented as PCoA based on Bray–Curtis distances. (**c**) PERMANOVA analysis (^◊◊^*P*<0.01, 999 permutations).

The alpha diversity of gut microbiota in mice was assessed using the Shannon index (Fig. 2a). The results of alpha diversity analysis revealed that no significant differences were found between the NM0 and the CIH0 groups. However, the Shannon index of the CIH6 group was lower than that of the NM6 group, and the results of Student’s t-test indicated that the difference between the two groups was statistically significant (Cohen’s d=1.072, *P*=0.029), suggesting a reduction in alpha diversity of gut microbiota in mice after 6 weeks of CIH. PCoA was employed to evaluate the beta diversity of gut microbiota. The PCoA analysis based on the Bray–Curtis dissimilarity was conducted for faecal samples before and after NM and CIH (Fig. 2b). Additionally, PERMANOVA analysis was conducted using the Bray–Curtis distance metric with 999 permutations (Fig. 2c). The results revealed that the projection distances of samples on the vertical coordinates were relatively close between the NM0 and CIH0 groups (Fig. 2b), and the results of the PERMANOVA analysis were pseudo-F=2.529, R^2^=0.123, adjusted *P*=0.002 (Fig. 2c). In contrast, for the comparisons of NM0 VS NM6, CIH0 VS CIH6 and NM6 VS CIH6, the projection distances of the samples on the vertical coordinate were relatively far (Fig. 2b). The corresponding PERMANOVA results were pseudo-F=22.957, R^2^=0.544, adjusted *P*=0.0012 (Fig. 2c); pseudo-F=19.979, R^2^=0.510, adjusted *P*=0.0012 (Fig. 2c); and pseudo-F=9.626, R^2^=0.337, adjusted *P*=0.0012 (Fig. 2c), respectively. These findings suggest that age is a significant factor influencing the beta diversity of gut microbiota. Furthermore, the results indicate that after 6 weeks of CIH treatment, the overall structure of the gut microbiota in mice exhibited substantial alterations. PCo1 and PCo2 in the PCoA explained 41.4% and 12.9% of the total variance of the microbiota, respectively.

### Analysis of the taxonomic composition of gut microbiota

16S rRNA gene sequencing was performed on the four groups of samples, NM0, NM6, CIH0 and CIH6, to demonstrate the relative abundance of gut microbiota at the ‘phylum’ and ‘genus’ levels, respectively.

**Fig. 3.**
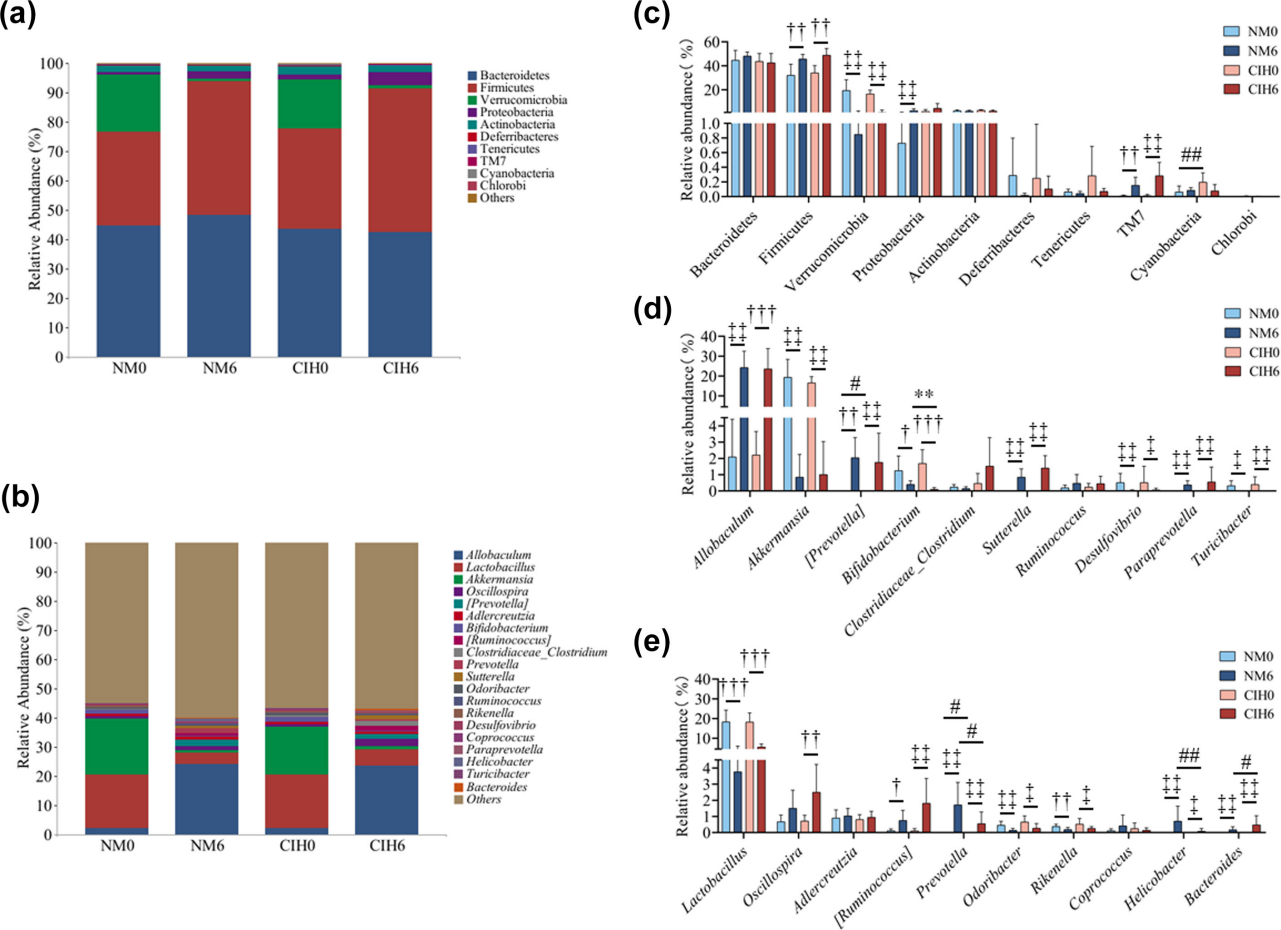
(**a**) Relative abundance of mouse gut microbiota at the phylum level. (**b**) Relative abundance of mouse gut microbiota at the genus level. (**c**) Comparison of composition and distribution of mouse gut microbiota at the phylum levels. (**d, e**) Comparison of composition and distribution of mouse gut microbiota at the genus levels. The results were presented as mean±sem. Statistical significance between the NM and CIH groups was assessed by Student’s t-test (^*^P<0.05 and ^**^P<0.01) or Mann–Whitney U test (^#^*P*<0.05 and ^##^*P*<0.01), while paired t-test (^†^*P*<0.05, ^††^*P*<0.01 and ^†††^*P*<0.001) or Wilcoxon signed-rank test (^‡^*P*<0.05 and ^‡‡^*P*<0.01) was employed to compare differences between the 0-week and 6-week intervention groups.

At the phylum level, the five phyla with the highest relative abundance in the four groups of samples, NM0, NM6, CIH0 and CIH6, are Bacteroidetes, Firmicutes, Verrucomicrobia, Proteobacteria and Actinobacteria (Fig. 3a). There was no significant change in the relative abundance of bacteria at the level of the top 5 phyla between the NM0 group and the CIH0 group (*P*>0.05) (Fig. 3c). Compared with the NM6 group, the relative abundance of Firmicutes and Proteobacteria in the CIH6 group increased, while the relative abundance of Bacteroidetes decreased, but the differences were not statistically significant (*P*>0.05) (Fig. 3c).

At the genus level, the top 10 genera by relative abundance in the NM0 group were *Akkermansia*, *Lactobacillus*, *Allobaculum*, *Bifidobacterium*, *Adlercreutzia*, *Oscillospira*, *Desulfovibrio*, *Odoribacter*, *Rikenella* and *Turicibacter*, which accounted for 19.37%, 18.35%, 2.10%, 1.25%, 0.91%, 0.67%, 0.52%, 0.46%, 0.38% and 0.32%, respectively (Fig. 3b). In the CIH0 group, the top 10 genera by relative abundance were *Lactobacillus*, *Akkermansia*, *Allobaculum*, *Bifidobacterium*, *Adlercreutzia*, *Oscillospira*, *Odoribacter*, *Acinetobacter*, *Rikenella* and *Desulfovibrio*, which accounted for 18.21%, 16.57%, 2.22%, 1.69%, 0.82%, 0.72%, 0.66%, 0.62%, 0.52% and 0.52%, respectively (Fig. 3b). Among the top 10 genera in the relative abundance in the NM0 and CIH0 groups, 9 genera were common, and the differences were not statistically significant (*P*>0.05) (Fig. 3d, e).

At the genus level, the top 10 genera by relative abundance in the NM6 group were *Allobaculum*, *Lactobacillus*, *[Prevotella]*, *Prevotella*, *Oscillospira*, *Adlercreutzia*, *Sutterella*, *Akkermansia*, *[Ruminococcus]* and *Helicobacter*, which accounted for 24.26%, 3.77%, 2.04%, 1.72%, 1.51%, 1.03%, 0.85%, 0.85%, 0.75% and 0.71%, respectively (Fig. 3b). In the CIH0 group, the top 10 genera by relative abundance were *Allobaculum*, *Lactobacillus*, *Oscillospira*, *[Ruminococcus]*, *[Prevotella]*, *(Clostridiaceae_Clostridium)*, *Sutterella*, *Akkermansia*, *Adlercreutzia* and *Prevotella*, which accounted for 23.56%, 5.57%, 2.50%, 1.82%, 1.76%, 1.53%, 1.41%, 1.01%, 0.95% and 0.55% (Fig. 3b). Compared with the NM6 group, the relative abundance of *Bacteroides* (rank-biserial=0.558, *P*=0.014) in the CIH6 group was significantly increased, while the relative abundance of *Bifidobacterium* (Cohen’s d=1.779, *P*=0.002), *Helicobacter* (rank-biserial=0.609, *P*=0.007) and *Prevotella* (rank-biserial=0.541, *P*=0.017) was significantly decreased, with statistically significant differences (*P*<0.05) (Fig. 3d, e).

The research results revealed that some phyla and genera in both NM and CIH groups demonstrated similar trends. For example, at the phylum level, the relative abundance of TM7 (Cohen’s d=−1.759, *P*=0.002; rank-biserial=0.886, *P*=0.006), Firmicutes (Cohen’s d=−1.911, *P*=0.002; Cohen’s d=−2.525, *P*=0.001) and Verrucomicrobia (rank-biserial=0.886, *P*=0.006; rank-biserial=0.886, *P*=0.006) exhibited significant changes in both NM and CIH groups before and after the intervention, with statistical significance (*P*<0.05) (Fig. 3c). Similarly, at the genus level, the relative abundance of *Bifidobacterium* (Cohen’s d=1.267, *P*=0.016; Cohen’s d=2.379, *P*=0.0002), *Prevotella* (rank-biserial=0.886, *P*=0.006; rank-biserial=0.886, *P*=0.006), *Rikenella* (Cohen’s d=1.429, *P*=0.003; rank-biserial=0.693, *P*=0.032), *Odoribacter* (rank-biserial=0.822, *P*=0.011; rank-biserial=0.661, *P*=0.041), *[Prevotella]* (Cohen’s d=−1.961, *P*=0.0006; rank-biserial=0.886, *P*=0.006), *Lactobacillus* (Cohen’s d=3.448, *P*=0.0001; Cohen’s d=3.853, *P*=4.78E-05), *Turicibacter* (rank-biserial=0.844, *P*=0.014; rank-biserial=0.886, *P*=0.006), *[Ruminococcus]* (Cohen’s d=−1.423, *P*=0.012; rank-biserial=0.886, *P*=0.006), *Allobaculum* (rank-biserial=0.886, *P*=0.006; Cohen’s d=−2.801, *P*=0.0001), *Sutterella* (rank-biserial=0.886, *P*=0.006; rank-biserial=0.886, *P*=0.006), *Desulfovibrio* (rank-biserial=0.886, *P*=0.006; rank-biserial=0.661, *P*=0.041), *Helicobacter* (rank-biserial=0.886, *P*=0.006; rank-biserial=0.843, *P*=0.014), *Akkermansia* (rank-biserial=0.886, *P*=0.006; rank-biserial=0.886, *P*=0.006), *Paraprevotella* (rank-biserial=0.886, *P*=0.006; rank-biserial=0.886, *P*=0.006) and *Bacteroides* (rank-biserial=0.822, *P*=0.011; rank-biserial=0.886, *P*=0.006) also exhibited significant changes, with statistical significance (*P*<0.05) (Fig. 3d, e).

### Analysis of microbial composition differences and marker microbes after 6 weeks of CIH exposure

#### Analysis of ASV results

A total of 9,988 ASVs were identified in the NM6 group, while 8,711 ASVs were identified in the CIH6 group. Of these, 8,408 ASVs were unique to the NM6 group, and 7,131 ASVs were unique to the CIH6 group. Additionally, there were 1,580 ASVs shared between the two groups.

#### LEfSe analysis

**Fig. 4.**
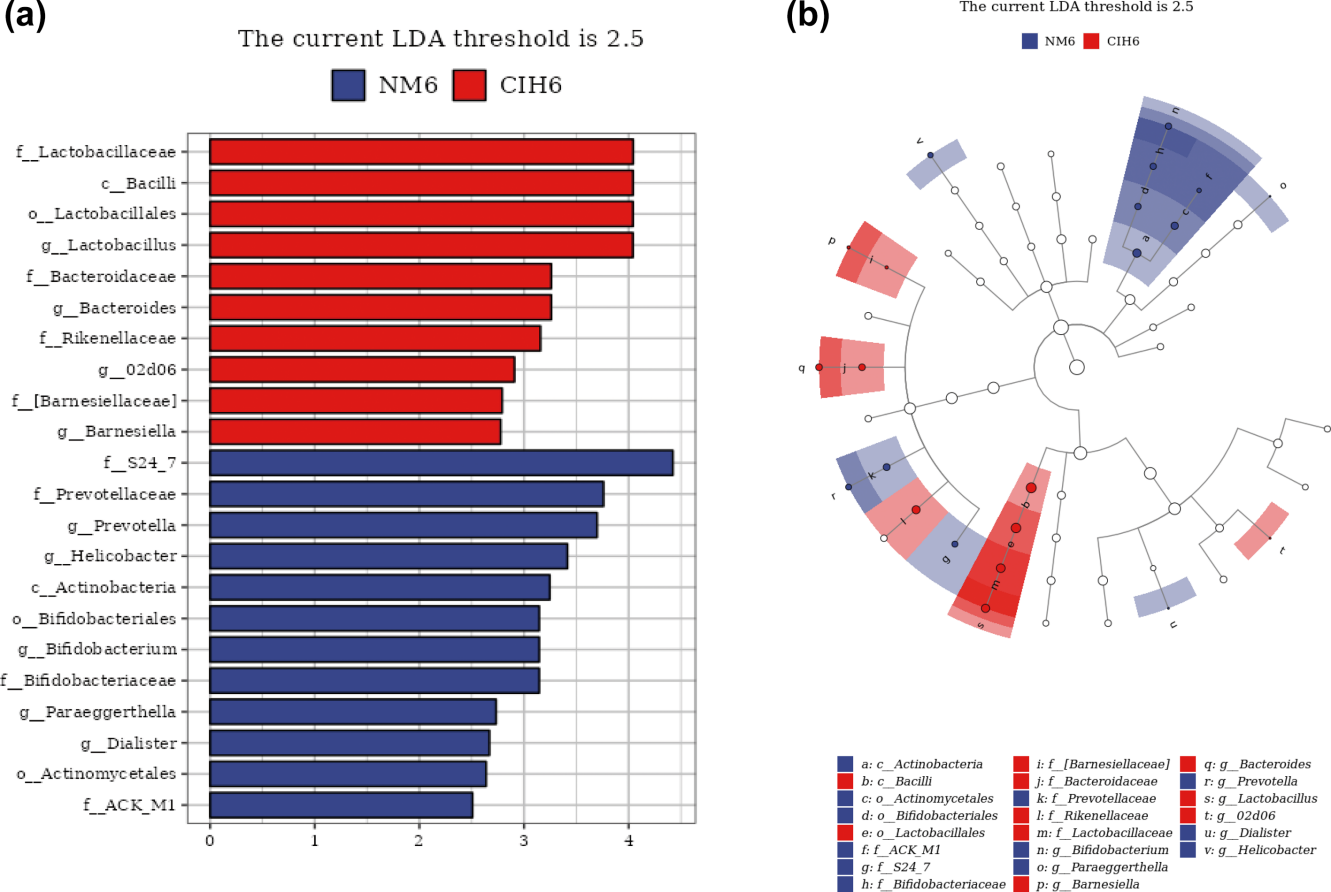
LEfSe analysis of gut microbiota in the NM6 and CIH6 groups. (**a**) Histogram of LDA score (LDA>2.5, *P*<0.05), where the vertical axis represents species with significant differences (*P*<0.05). Red indicates a higher abundance of the species in the CIH6 group, while blue indicates a higher abundance of the species in the NM6 group. (**b**) Dendrogram, with red nodes indicating species with significant differences (*P*<0.05) and higher abundance in the CIH6 group, blue nodes indicating species with significant differences (*P*<0.05) and higher abundance in the NM6 group and hollow nodes indicating species with no significant difference (*P*>0.05). Letters denote the names of species with significant differences (*P*<0.05).

In order to identify the gut microbiota closely associated with CIH, LEfSe analysis was utilized to discern the key gut microbiota responsible for distinguishing between the NM6 and CIH6 groups at the levels of phylum, class, order, family and genus (Fig. 4). The LDA results revealed 22 bacterial taxa that exhibited significant differences between the two groups (LDA>2.5, *P*<0.05). In the NM6 group, 12 bacterial genera were significantly different and prominently enriched in this group (LDA>2.5, *P*<0.05), predominantly composed of Actinobacteria at the class level; Bifidobacteriales and Actinomycetales at the order level; S24_7, Prevotellaceae, Bifidobacteriaceae and ACK_M1 at the family level; and *Prevotella*, *Helicobacter*, *Bifidobacterium*, *Paraeggerthella* and *Dialister* at the genus level. Conversely, in the CIH6 group, ten bacterial genera exhibited significant differences and were distinctly enriched in this group, predominantly composed of Bacilli at the class level; Lactobacillales at the order level; Lactobacillaceae, Bacteroidaceae, Rikenellaceae and [Barnesiellaceae] at the family level; and *Lactobacillus*, *Bacteroides*, *02d06* and *Barnesiella* at the genus level. These findings suggested that CIH altered the key microbe of the gut microbiota in mice after 6 weeks.

#### Analysis of marker microbe

**Fig. 5.**
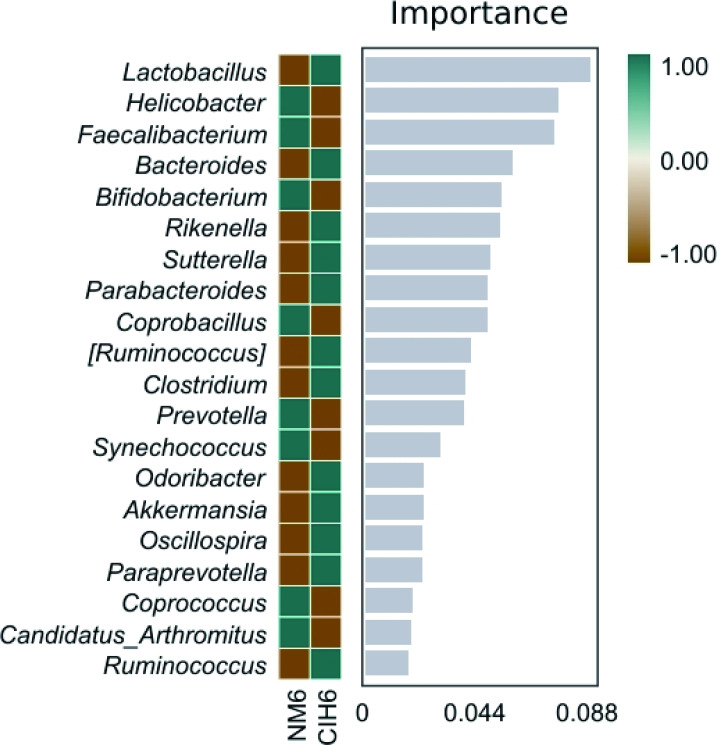
Random forest analysis. The heat map illustrated the abundance distribution of these species in each group. The significance of species to the model decreases progressively from top to bottom.

The results of the random forest model analysis revealed that *Lactobacillus*, *Helicobacter*, *Faecalibacterium*, *Bacteroides*, *Bifidobacteriaceae*, *Rikenella*, *Sutterella*, *Parabacteroides*, *Coprobacillus* and *[Ruminococcus]* were among the top 10 important genera for distinguishing between the NM6 and CIH6 groups, with *Lactobacillus* being the most important in discriminating between the 2 groups (Fig. 5).

#### Functional prediction analysis

**Fig. 6.**
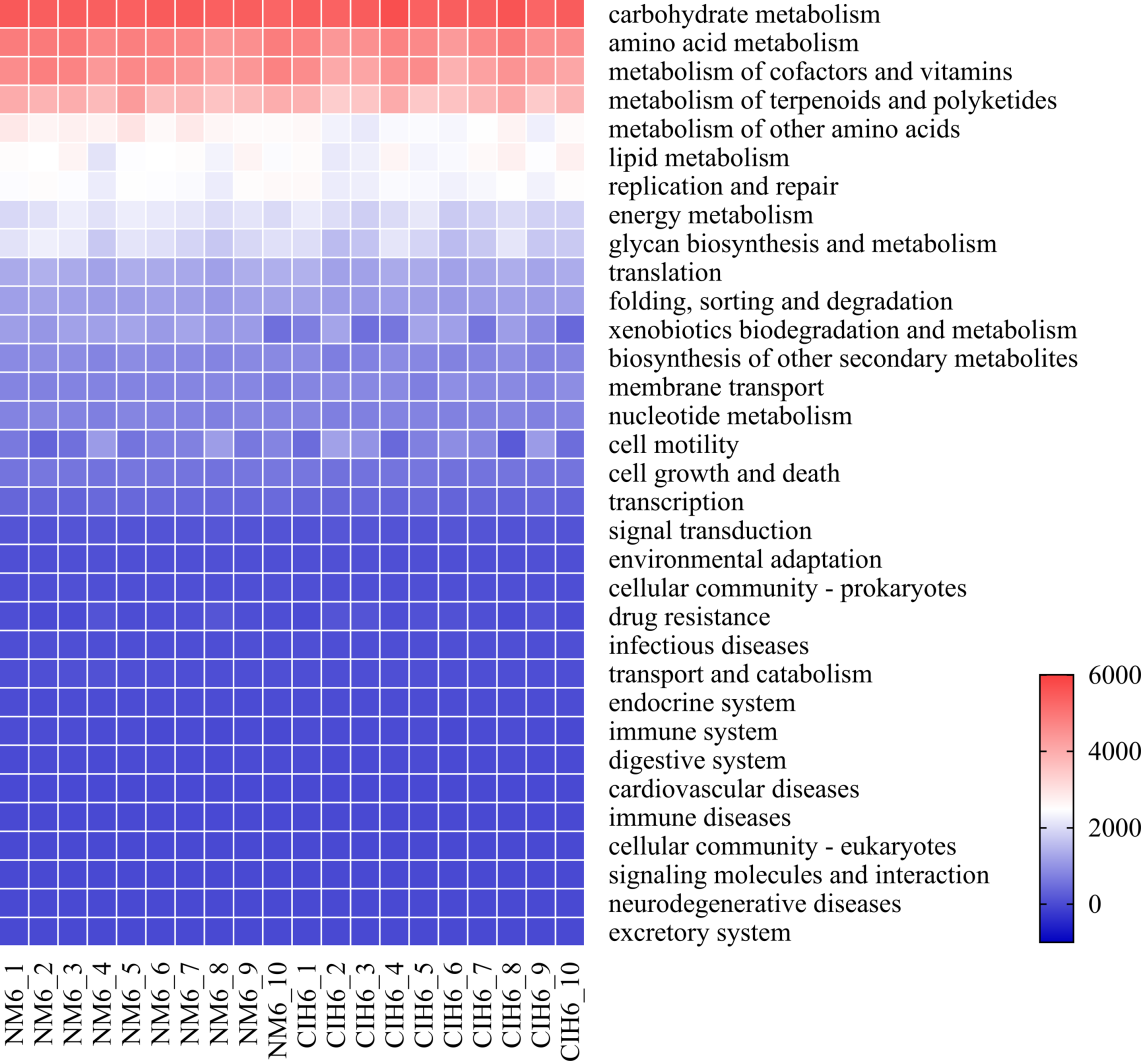
Heatmap illustrating KEGG secondary metabolic pathways in the NM6 and CIH6 groups.

Predictive functional analysis of the gut microbiota in the NM6 and CIH6 groups was conducted using PICRUSt2. Based on the abundance analysis results of metabolic pathways obtained from the KEGG database, a total of 33 biological metabolic pathway functions were identified in the two groups at the secondary metabolic pathway level, which could be categorized into 6 classes according to level 1. These include 5 categories of cellular processes, 3 categories of environmental information processing, 4 categories of genetic information processing, 5 categories of human diseases, 11 categories of metabolism and 5 categories of organismal systems, with metabolism emerging as the most critical pathway. A heatmap was generated at the secondary level of metabolic pathways, as depicted in Fig. 6, and each sample was mainly enriched in carbohydrate metabolism, amino acid metabolism, metabolism of cofactors and vitamins and metabolism of terpenoids and polyketides.

**Fig. 7.**
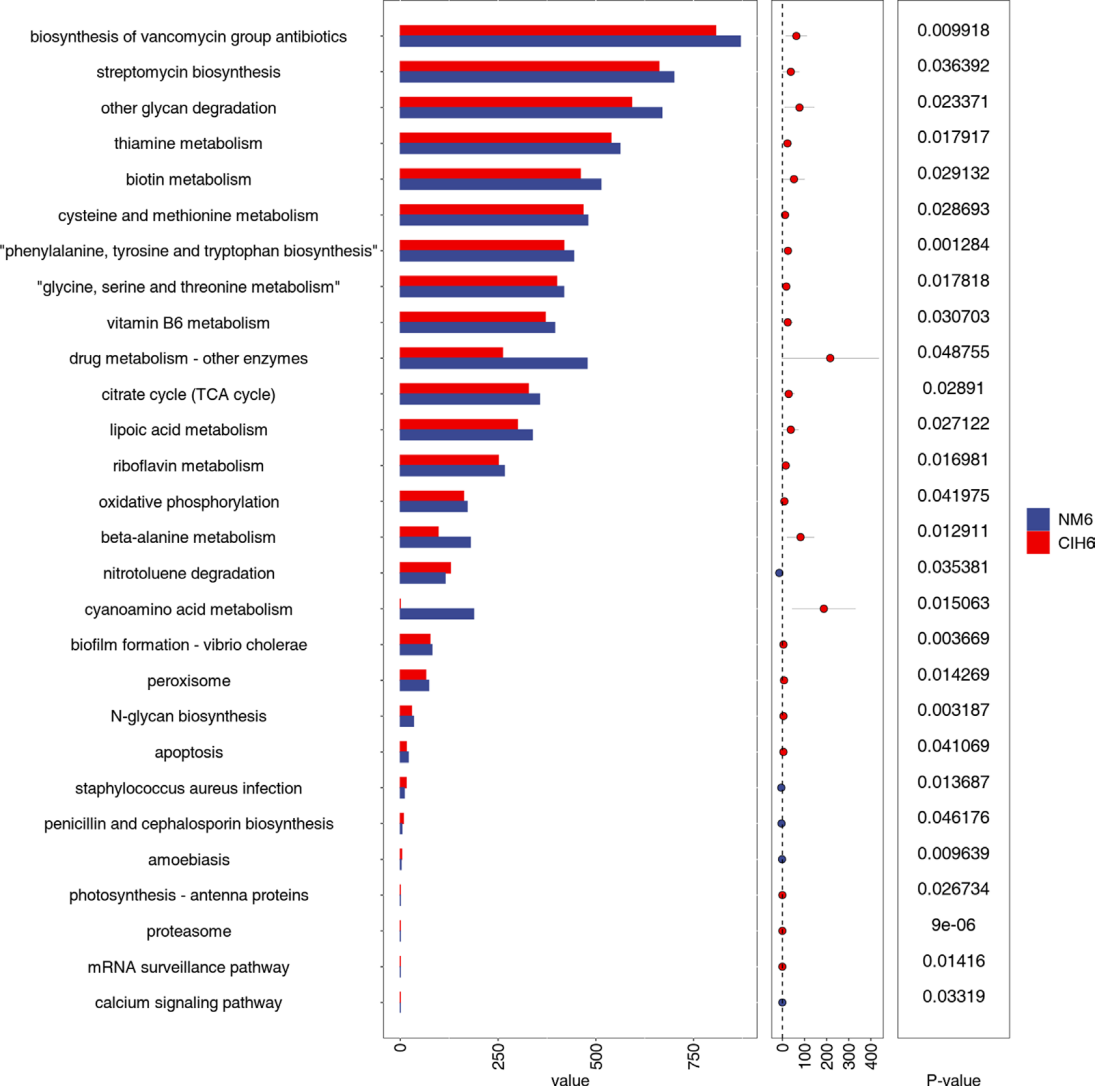
Comparative analysis of KEGG tertiary metabolic pathways between the NM6 and CIH6 groups, with results presented as mean±sd.

At the tertiary metabolic pathway level, the NM6 and CIH6 groups collectively obtained 174 gene metabolic pathways. Differential expression of metabolic pathways in the NM6 and CIH6 groups at the tertiary level was assessed using Student’s t-test combined with the Benjamini–Hochberg FDR correction method (*FDR*<0.05). A total of 28 metabolic pathways exhibited differential expression between the NM6 and CIH6 groups at the tertiary level (Fig. 7). Compared to the NM6 group, the CIH6 group showed significant reductions in biosynthesis of vancomycin group antibiotics, streptomycin biosynthesis, other glycan degradation, biotin metabolism, drug metabolism – other enzymes, lipoic acid metabolism, beta-alanine metabolism and cyanoamino acid metabolism, and these differences were statistically significant (*P*<0.05).

## Discussion

Since the repetitive collapse of the airway leading to intermittent hypoxia-reoxygenation events is one of the main manifestations of OSAS [17], this study simulated CIH events, resulting from airway collapse during sleep in OSAS patients by exposing mice to a hypoxic environment, thereby establishing an OSAS mouse model. The impact of CIH on the composition and abundance of gut microbiota was assessed through 16S rRNA sequencing. Differences in gut microbiota between mice exposed to NM for 6 weeks and those subjected to CIH for 6 weeks were compared, and the functional analysis of gut microbiota was performed using PICRUSt, based on the KEGG database. The study revealed differences in the abundance, composition and functionality of gut microbiota between the two groups of mice.

Currently, there is no consensus on the alterations in gut microbiota diversity in the OSAS mouse model. Moreno-Indias *et al.* suggested that CIH increased the alpha diversity of the gut microbiota (Shannon index, *P*=0.001) [18], a finding corroborated by Zhang *et al.* [19]. However, other researchers have reported that CIH reduced the alpha diversity of the gut microbiota (Shannon index, *P*=0.0046) [20]. The results of this study indicate that before intervention, there was no statistically significant difference in the alpha and beta diversity of gut microbiota between the NM0 and CIH0 groups. However, after 6 weeks, the Shannon index of CIH mice showed a decreasing trend, suggesting that hypoxia may lead to a reduction in the diversity of gut microbiota.

This study revealed that at the phylum level, the phyla exhibiting the highest relative abundance in the NM6 and CIH6 groups were consistent, including Bacteroidetes, Firmicutes, Verrucomicrobia, Proteobacteria and Actinobacteria. Compared with the NM6 group, the relative abundance of Firmicutes and Proteobacteria in the CIH6 group was increased, while the abundance of Bacteroidetes decreased, which is similar to the findings of Wang *et al.* [2]. Firmicutes utilize dietary fibre to produce short-chain fatty acids (SCFAs) such as acetate and butyrate, thereby regulating gut homeostasis, inflammatory factors, glucose metabolism and lipid metabolism, playing a crucial role in maintaining individual health [21]. Bacteroidetes was also recognized as a beneficial component of the gut microbiota, playing a crucial role in polysaccharide degradation [22]. The observed alterations in the relative abundances of Firmicutes and Bacteroidetes indicate that CIH can disrupt the composition of the gut microbiota in individuals.

In this study, after 6 weeks of CIH, the relative abundance of *Bacteroides* significantly increased, while the abundance of *Bifidobacterium*, *Helicobacter* and *Prevotella* significantly decreased. Both LEfSe analysis and random forest model analysis indicated that these genera were key gut microbiota with significant differences between the NM6 and CIH6 groups, serving as important genera for discriminating between NM and CIH. Previous studies have suggested that CIH can decrease the abundance of beneficial genera such as *Bacteroides* and increase the abundance of *Bifidobacterium*. [23]. Consistent with these findings, Zhang *et al.* reported an increase in the abundance of *Bacteroides* and a decrease in the abundance of *Bifidobacterium* after 6 weeks of CIH [19]. *Bacteroides* is a major producer of SCFAs in the gut, mainly producing acetate and propionate, which contribute to maintaining gut homeostasis [24]. The increase in such bacteria may result from organism adaptation. *Bifidobacterium* is a key symbiotic bacterium in the gut, capable of producing vitamins, acetate and antimicrobial substances, thereby improving epithelial cell-mediated intestinal defence and protecting the host from fatal infections [25]. Our results also showed a decrease in the abundance of *Prevotella*, a beneficial genus with high fibre utilization ability, which belongs to the propionate-producing genera [26]. Researchers analysing the gut microbiota of patients with OSAS combined with hypertension found that the relative abundance of *Prevotella* in the gut was lower than that in patients with hypertension alone [27], suggesting that CIH may be an important factor in such changes in microbial composition. SCFAs are primarily produced by microbial fermentation of dietary fibre in the gut, including acetate, propionate and butyrate, and they play a role in protecting the intestinal barrier [9]. Feng *et al.* cultured Caco-2 intestinal epithelial cells *in vitro* to simulate the intestinal barrier and found that SCFAs inhibit the activation of NLRP3 inflammasome and autophagy induced by LPS, stimulate the formation of tight junctions and thereby reduce intestinal permeability [28]. Li *et al.* found that the levels of intestinal barrier biomarkers intestinal fatty acid-binding protein and d-lactate in the plasma of OSAS patients were significantly increased, indicating damage to intestinal epithelial cells and increased intestinal mucosal permeability [29], possibly related to CIH events experienced by OSAS patients. This study’s results indicate that after 6 weeks of exposure to a CIH environment, the gut microbiota of mice became dysregulated, with an altered structure of SCFA-producing microbiota, suggesting that changes in gut microbiota structure induced by CIH may be involved in alterations in intestinal barrier function, and their relationship merits further exploration.

This study utilized PICRUSt2 to predict the functional disparities in microbial communities between the NM and CIH groups. The functional prediction identified 28 third-level pathways associated with CIH (*P*<0.05), with metabolism emerging as the principal functional pathway. The CIH6 group exhibited significant reductions in pathways such as lipoic acid metabolism, beta-alanine metabolism and cyanoamino acid metabolism, suggesting that CIH may disrupt gut environmental homeostasis through metabolic signalling pathways. Previous studies have demonstrated that lipoic acid supplementation restored NO levels in CIH mice, upregulated the expression of antioxidant enzyme genes and concurrently downregulated levels of endogenous competitive antagonists of NOS (ADMA), inflammatory markers (TNF-α) and oxidative stress markers (8-OHdG, 8-isoprostane), thereby preserving cardiovascular and renal function [30,31]. The functional prediction results of this study indicate a decline in lipoic acid metabolism after 6 weeks of CIH, further suggesting that gut microbiota may play a role in the pathogenesis of comorbidities associated with OSAS by modulating intestinal metabolic functions.

This study has limitations. Although this study analysed the diversity and composition of gut microbiota before intervention and found that the two groups had similar baselines, and this study also excluded age [32] as a confounding factor affecting the composition of gut microbiota, this study cannot rule out the influence of genetic factors [33] on gut microbiota. Gender may influence the composition and function of gut microbiota [34]. However, this study was solely focused on analysing the changes in the gut microbiota of male mice. As a result, it remains unclear whether CIH elicits similar alterations across different genders. This study utilized PICRUSt2 to predict the biological functions of microbial communities. However, this predictive method still has limitations due to the limited availability of bacterial isolates obtained, and the relationship between CIH and gut microbiota remains unclear. The use of an earlier version of the Greengenes database could potentially impact the accuracy and comprehensiveness of our results. We acknowledge the limitations of our current statistical approach and emphasize the need for caution in interpreting the results presented in Fig. 3(c–e). Furthermore, this study solely focused on alterations in the gut microbiota of mice without delving into the effects of CIH on gut metabolites, the integrity of the mouse intestinal barrier and blood-related indicators. Future research endeavours should incorporate metabolomics, transcriptomics and other methodologies to delve deeper into these mechanisms.

## Conclusion

In conclusion, this study further confirms the influence of CIH exposure on the gut microbiota of mice, identifying several gut microbiota genera producing SCFAs that were associated with CIH exposure. These findings offer a theoretical foundation for further research on restoring gut microbiota, targeting gut microbiota metabolites or developing specific microbial communities for the prevention or treatment of CIH.

## Supplementary material

10.1099/jmm.0.002069 Uncited Supplementary Material 1.

## Abbreviations

ASV: amplicon sequence variant
CIH: chronic intermittent hypoxia
DADA2: Divisive Amplicon Denoising Algorithm 2
KEGG: Kyoto Encyclopedia of Genes and Genomes
LDA: linear discriminant analysis
LEfSe: linear discriminant analysis effect size
NM: normoxia
OSAS: obstructive sleep apnoea syndrome
PCoA: principal coordinate analysis
PICRUSt2: Phylogenetic Investigation of Communities by Reconstruction of Unobserved States 2
SCFAs: short-chain fatty acids
SPF: specific-pathogen-free
